# Cooking Methods for Preserving Isothiocyanates and Reducing Goitrin in *Brassica* Vegetables

**DOI:** 10.3390/foods12193647

**Published:** 2023-10-02

**Authors:** Thanaporn Panduang, Pakkapong Phucharoenrak, Weeraya Karnpanit, Dunyaporn Trachootham

**Affiliations:** 1 Master of Science Program in Toxicology and Nutrition for Food Safety, Institute of Nutrition, Mahidol University, Nakhon Pathom 73170, Thailand; thanaporn.pad@student.mahidol.ac.th; 2 Institute of Nutrition, Mahidol University, Nakhon Pathom 73170, Thailand; pakkapong.phu@mahidol.ac.th; 3 School of Science, Western Sydney University, Locked Bag 1797, Penrith, NSW 2751, Australia; w.karnpanit@westernsydney.edu.au

**Keywords:** cooking, *Brassica* vegetables, goitrogen, goitrin, isothiocyanates, benzyl isothiocyanate, sulforaphane, LC-MS/MS

## Abstract

Glucosinolates in *Brassica* vegetables can be hydrolyzed into various products, e.g., chemopreventive agents, isothiocyanates (ITCs) and anti-thyroid substance, goitrin. Cooking can reduce goitrin but destroy isothiocyanates. This study aimed to optimize cooking conditions for reducing goitrin while preserving isothiocyanates in *Brassica* vegetables. Cabbage and Chinese kale samples were divided evenly into raw, blanched, steamed, and water-based stir-fried samples. Cooking temperature and time were varied at 60, 80, or 100 °C for 2, 4, or 6 min. The levels of goitrin, benzyl isothiocyanate (BITC), and sulforaphane (SFN) were measured using LC-MS/MS. Response surface model (RSM) was used to identify the optimal cooking conditions to reduce goitrin but preserve ITCs. Results showed that goitrin content in cabbage depended on the cooking methods, temperature, and time, while that of Chinese kale only depended on the methods. In contrast, the concentrations of SFN in cabbage and BITC in kale depended on the cooking temperature and time but not methods. Based on RSM analysis, the suggested household cooking methods for preserving isothiocyanates and reducing goitrin are steaming cabbage at 80–100 °C for 4 min and stir-frying Chinese kale at 60–100 °C for 2 min. Such methods may preserve the bioactive compounds while reducing food hazards.

## 1. Introduction

*Brassica* vegetables, or cruciferous vegetables, e.g., cabbage, Chinese kale, cauliflower, brussels sprouts, watercress, red cabbage, bok choy, and broccoli have two main characteristics: a bitter taste and a sulfurous smell [1]. Such unique properties are derived from a family of phytochemicals, isothiocyanates (ITCs). ITCs are metabolic products obtained from the hydrolysis of various types of glucosinolates. Once the vegetables are chopped, cooked with heat, or chewed in the mouth, the myrosinase enzyme will be activated, catalyzing the generation of ITCs via a hydrolysis reaction [2]. ITCs including beta-phenethyl isothiocyanate (PEITC), sulforaphane, and benzyl isothiocyanate (BITC) were well documented for cancer chemopreventive effects [3,4]. Several previous studies have found that ITCs can inhibit the development of cancer in a variety of organs in animal models, including oral, prostate, breast, colon, lung, gastric, and liver [5,6,7]. Clinical trials showed that ITCs can promote detoxification and urinary excretion of tobacco-derived and grilled meat-derived carcinogens [8,9,10]. Case–control and cohort studies also found that consumption of *Brassica* vegetables is associated with decreased risk of several types of cancer [11,12,13]. However, these vegetables also contain 2-hydroxy-3-butenyl glucosinolate (progoitrin) and indole glucosinolate, which can be converted to goitrin and thiocyanate, which act as goitrogens in animal models. The conversion can occur either via spontaneous cyclization or activated by the myrosinase enzyme [14,15]. These goitrogens may block iodide transport to the thyroid gland and inhibit the incorporation of iodine into thyroglobulin, impairing the synthesis of the active thyroid hormone. To compensate for the deficiency, thyroid-stimulating hormone (TSH) has been increasingly produced in the pituitary gland to stimulate more thyroid hormone production [16]. The increased TSH secretion was found to be associated with an increased risk of thyroid cancer [16]. A previous meta-analysis reported that the consumption of *Brassica* vegetables may be associated with an increased risk of thyroid cancer, especially in areas where iodine deficiency is commonly found [17,18]. A previous review suggested that the level of thiocyanate from human consumption of *Brassica* vegetables was extremely low with negligible adverse effects [14,19]. In contrast, the level of goitrin derived from the vegetable can be detectable and still be able to decrease iodine uptake by the thyroid gland [19]. Therefore, goitrin is still a goitrogenic substance of concern. Previous studies have shown that uncooked Chinese kale and cabbage contain high amounts of progoitrin, compared to other types of *Brassica* vegetables [19,20]. Since progoitrin can be converted to goitrin and cabbage and Chinese kale are popularly consumed worldwide, strategies to reduce goitrin in Chinese kale and cabbage are crucial. 

Some food preparation methods such as washing, soaking, and boiling were shown to help reduce goitrogens in food by destroying myrosinase and preventing the liberation of goitrin from progoitrin [14,15,20]. Unfortunately, heat can also destroy glucosinolate and isothiocyanates [21,22,23]. To date, no studies have been conducted to identify which cooking condition is the best for reducing goitrin, while preserving isothiocyanates in *Brassica* vegetables. A previous study showed that moist heat methods can reduce progoitrin with blanching being the best one, compared with boiling and steaming [22]. However, cooking with high heat for a long duration such as boiling can destroy ITCs [22,23]. Therefore, we hypothesize that the best method to reduce goitrin while preserving ITCs should be moist heat with short duration. This study aimed to optimize household cooking conditions to preserve functional substances like ITCs while also reducing anti-thyroid substances like goitrin. The effect of cooking methods via moist heat including steaming, blanching, and water-based stir-frying, cooking time, and temperature were studied. Among the isothiocyanates, sulforaphane and BITC have been found in significant amounts in cabbage and Chinese kale, respectively. Furthermore, these compounds’ chemopreventive effects against various types of cancer are well documented [24,25]. Thus, in this paper, we explore the cooking conditions to reduce goitrin while preserving SFN and BITC.

The insight from this work may be used for the recommendation of household cooking methods to help reduce hazardous chemicals and preserve the beneficial substances in *Brassica* vegetables.

## 2. Materials and Methods

### 2.1. Materials, Reagents, and Standards

Cabbage (*Brassica oleracea* L. var. *capitata* L.) and Chinese kale (*Brassica alboglabra* L.H. Bailey) were purchased from Salaya market, Phutthamonthon District, Nakhon Pathom Province, Thailand. Benzyl isothiocyanate standard (Purity: 98%) and (R)-sulforaphane standard (Purity: 98%) were products of Toronto Research Chemicals (Toronto, ON, Canada), which were purchased from Phoenix Scientific Co., Ltd. (Bangkok, Thailand). Goitrin standard (Purity: 98%) was purchased from Wuhan ChemFaces Biochemical Co., Ltd. (Wuhan, China). Ammonia (2 M) in methanol was a product of AcroSeal™ (Thermo Scientific Chemicals, Somerville, NJ, USA). Formic acid, dimethyl sulfoxide (DMSO), and liquid chromatography-mass spectrometry (LC-MS) grade of methanol and acetonitrile (ACN) were products of Merck Millipore (Merck KGaA, Darmstadt, Germany. Hexane was a product of J.T. Baker Chemical (Phillipsburg, NJ, USA). LC-MS grade of acetonitrile was a product of RCI Labscan Ltd. (Bangkok, Thailand). Type I grade water was obtained freshly from a Purelab Quest UV water purifying system (Elga LabWater, High Wycombe, UK). 

### 2.2. Cooking Conditions and Sample Collections

**Figure 1:**
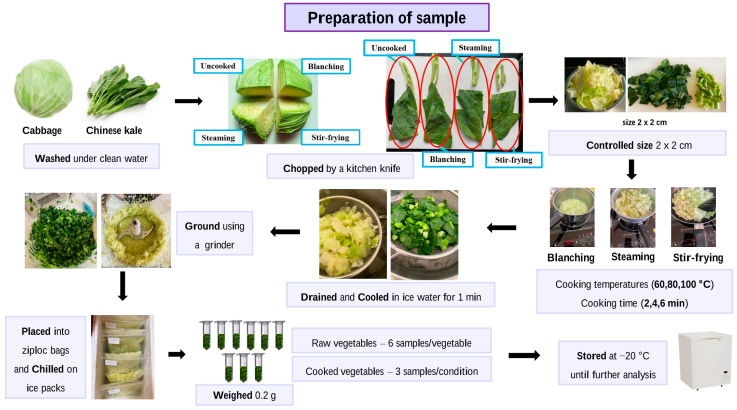
Preparation of cabbage and Chinese kale samples using various cooking conditions.

Sample preparation was performed according to previous studies [20,22] with some modifications. Cabbage and Chinese kale were purchased from the Salaya market, Nakhon Pathom, Thailand. Each vegetable was randomly selected. The cabbage was rinsed with clean water and chopped into four equal parts, as shown in Figure 1. Each part was used for uncooked, blanching, steaming, and stir-frying samples. For Chinese kale, both leaf and stalk were used, rinsed with clean water, and cut into four equal parts for uncooked, blanching, steaming, and stir-frying samples (Figure 1).

Each divided part of the vegetable was chopped into 2 × 2 cm pieces before cooking. The samples were processed using different cooking methods, i.e., blanching, steaming, and stir-frying under specific cooking temperatures (60, 80, and 100 °C) and cooking times (2, 4, and 6 min) conditions. All cooking conditions were performed using the same induction cooker (Touch type Induction Cooker, SEAGULL touch set, SEAGULL^®^, Bangkok, Thailand) and the same saucepan and pot. The detailed cooking condition for each cooking method is shown in Table 1. 

Uncooked (raw) vegetables were used as control samples. After cooking, the cooked vegetables were drained and cooled in ice water (with 1000 g ice and 2000 g water) for 1 min to stop the heat-induced hydrolysis reaction. Each cooked or uncooked sample was ground separately using a grinder (OTTO^®^, Bangkok, Thailand). After that, each sample was placed immediately into Ziploc bags and chilled on ice packs in polystyrene containers for transport from the kitchen to the lab. Three samples (0.2 g each) of each cooking condition were randomly collected into a 2 mL micro-centrifuge tube. For the uncooked vegetables, 6 samples were collected. All samples were stored at −20 °C in the freezer until further analysis. Each sample has been prepared separately before analysis. Therefore, the mean ± standard deviation displayed in the table is derived from n = 6 for uncooked vegetables and n = 3 for each cooking condition.

### 2.3. Sample Preparation for Determination of Goitrin, BITC, and Sulforaphane in Cooked and Uncooked Vegetables 

The sample preparation was performed according to previous studies [9,26,27] with some modifications. Hydrolysis and hexane extraction were performed similarly for all substances. Three randomly selected samples of cooked vegetables and six randomly selected samples of uncooked vegetables were collected. Frozen vegetable samples (0.2 g) were mixed with 1 mL of PBS (1:5) and mixed on a high-speed vortex for 30 s. To stimulate hydrolysis and release goitrins, BITC, and SFN, the mixtures were incubated for three hours in a water bath at 37 °C. Then, 300 µL hexane was added to each sample using a positive displacement pipette (Mettler-Toledo Rainin LLC, Oakland, CA, USA), and mixed on a high-speed vortex for 30 s. Subsequently, 2000 g samples were centrifuged at 25 °C for 3 min, and 200 µL of the clear supernatants of samples were collected into a new 2 mL microcentrifuge tube. Hexane extraction was performed twice and combined with the first extract. Ammonia derivatization was performed before the analysis of goitrin and BITC. Briefly, 400 µL of 2 M ammonia in methanol was added to the hexane extract and mixed for 30 s on a vortex. Then, the samples were incubated at room temperature on a shaker for 3 h followed by 4 °C overnight. The samples were dried in a SpeedVac concentrator (Labconco, Kansas City, MO, USA) at 55 °C for 40 min to remove the solvents and stored at −20 °C until further analysis. The dried samples were dissolved with 250 µL of 60% ACN (acetonitrile: water in a 3:2 ratio) and mixed on vortex for 1 min. Then, the samples were filtered through a 0.2 μm nylon filter before LC-MS/MS analysis. For the analysis of SFN, a non-derivatized method was used. For SFN, samples were prepared without derivatization. Briefly, the hexane extract was dried in a SpeedVac concentrator at 55 °C for 40 min to remove the solvents and stored at −20 °C until further analysis. The dried samples were dissolved with 200 µL of 60% ACN (acetonitrile: water in a 3:2 ratio) and mixed on vortex for 1 min. Then, the samples were filtered through a 0.2 μm of nylon filter for LC-MS/MS analysis. 

### 2.4. Preparation of Standard Concentrations of Goitrin-NH_3_, BITC-NH_3_, and SFN for Standard Calibration Curves

Preparation of the standard of goitrin-NH_3_, BITC-NH_3_, and SFN for calibration curves was performed according to a previous study [9,26,27] with some modifications. Calibration curves of BITC-NH_3_, goitrin-NH_3_, and SFN were used for calculation. The concentrations used to generate the calibration curve for BITC included 0.1, 0.5, 1, 5, 10, and 50 ng/mL. Those of goitrin-NH_3_ and SFN included 0.1, 0.5, 1, 5, 10, 50, 100, 250, and 500 ng/mL. The standard stock solution of goitrin (4 mg/mL) was prepared by dissolving 20 mg of goitrin in 5 mL of DMSO. Then, the solution was further diluted with ACN to the working concentrations. The standard stock solution of BITC (1 mg/mL) was prepared by dissolving 1 μL of BITC standard in 1.125 mL of methanol. The solution was further diluted with methanol to the working concentrations. All standard solutions of goitrin and BITC had an equal final volume of 200 µL. For each standard of BITC and SFN, 140 µL of 2 M ammonia in methanol was added to derivatize and mixed for 30 s on a vortex. The standard mixtures were incubated at room temperature on a shaker for 3 h, followed by 4 °C overnight. The mixtures were dried in a SpeedVac concentrator at 55 °C for 40 min to remove the solvents. The dried samples were stored at −20 °C. On the day of analysis, the dried samples were dissolved with 200 µL of 60% ACN (ACN: water in a 3:2 ratio) and mixed on vortex for 1 min. Then, the standard solution at each concentration of goitrin-NH_3_ or BITC-NH_3_ was placed in a glass vial with an insert for LC-MS/MS analysis. 

The standard stock solution of SFN (10 mg/mL) was prepared by dissolving 10 mg of SFN in 1 mL of methanol. The solution was further diluted with methanol to the working concentrations at the volume of 200 µL. Then, the standard was put in a glass vial with an insert for the LC-MS/MS analysis.

### 2.5. Determination of the Goitrin, BITC, and SFN in Samples Using LC-MS/MS Assay

LC-MS/MS analysis was performed according to a previous study [9] with some modifications. Measurement of goitrin and BITC was performed simultaneously, while measurement of SFN was performed in different injections but in the same batch of run as those of goitrin and BITC. The extracts were analyzed via the Ultimate 3000 Ultra High-performance liquid chromatography (UHPLC) system (Thermo Scientific, Waltham, MA, USA) using Hypersil GOLD^TM^ C18 column with a 100 × 2.1 mm, 1.9 m particle size, at a flow rate of 0.3 mL/min. The injection volume was 5 μL. The mobile phase consisted of 100% ACN (A) and 5 mM formic acid (B). For the determination of goitrin-NH_3_ and BITC-NH_3_, a gradient running program of 5.00 min was set as follows: 0–1.20 min, 50–50% B; 1.20–2.00 min, 50–75% B; 2.00–3.50 min, 75–75% B; 3.50–5.00 min, and 75–50% B. The retention time of BITC-NH_3_ and goitrin-NH_3_ were 1.246 and 1.019 min, respectively. For SFN analysis, an isocratic running program at 50:50 of the mobile phase A: B was run for 3.50 min. The retention time of SFN was 1.179 min.

Tandem mass spectrometric analysis was conducted using positive ion mode (ESI+) combined with an electron spray ionization probe at a spray voltage of 3500 V under the N_2_ sheath. Sheath gas had an arbitrary unit (Arb) value of 50 while auxiliary gas had a value of 10.0. Temperatures for the vaporizing and ion transfer tubes (ITT) were 325 °C and 350 °C, respectively. For simultaneous analysis of multiple masses, the mass spectrometer analysis was conducted via the selected reaction monitoring (SRM) mode. The mass-to-charge ratio of goitrin-NH_3_ precursor, quantified, and confirmation product ions were 146.85 *m*/*z*, 126.49 *m*/*z* (transition collision energy at 5.25 V), and 105.97 *m*/*z* (transition collision energy at 5.34 V), respectively. The mass-to-charge ratio of BITC-NH_3_ precursor, quantified, and confirmation product ions were 167 *m*/*z*, 91.125 *m*/*z* (transition collision energy at 17.43 V), and 65.042 *m*/*z* (transition collision energy at 39.5 V), respectively. The mass-to-charge ratio of SFN precursor, quantified, and confirmation product ions were 178 *m*/*z*, 144.07 *m*/*z* (transition collision energy at 10.48 V), and 72.04 *m*/*z* (transition collision energy at 26.57 V), respectively. Chromeleon™ Chromatography Data System (CDS) was used to acquire and analyze the LC-MS/MS data.

### 2.6. Method Validation

The validation of analytical methods was conducted according to the International Council for Harmonisation of Technical Requirements for Pharmaceuticals for Human Use (ICH) guidelines [28] and guidelines for standard method performance requirements of The Association of Official Analytical Chemists (AOAC) 2016 [29]. Performance characteristics of the method validation of the determination of goitrin, BITC, and SFN included linearity, precision, and accuracy. Calibration curves were created for linearity by plotting the peak area versus calibration standard concentration. The acceptable coefficient of determination (R^2^) is 0.995 or higher [30]. To estimate the inter-day precision, 10 replicates of vegetable samples spiked with goitrin, BITC, and SFN at a concentration of 1 ng/mL were analyzed on 10 different days. The percent of relative standard deviation (% RSD) was calculated. The acceptance criteria of % RSD are <21% at a concentration of 10 ng/mL and <30% at a concentration of 1 ng/mL [29]. The accuracy of the proposed method was examined as percentages of recovery. The standard solutions of goitrin, BITC, and SFN (10 ng/mL) were spiked to vegetable sample solutions in triplicate. The concentrations of the analytes were determined for the spiked samples. The acceptable recovery percentages were 60–115% at a concentration of 10 ng/mL [29]. 

### 2.7. Data Analysis

Graphing and statistical analyses were performed using GraphPad Prism version 9.5.1 (GraphPad Software, Inc., Boston, MA, USA) and Minitab Statistical Software version 21.1.0 (Minitab, Inc., State College, PA, USA). All data were shown as the mean ± standard deviation. The concentrations of goitrin, SFN, and BITC in uncooked and cooked vegetables were calculated using a linear regression equation generated from the respective standard curves. The dilution factor was applied when it was needed. Comparison of the goitrin, BITC, and SFN levels between cooked vegetables via different cooking methods under controlled different cooking temperatures and time (blanching, steaming, and stir-frying) and uncooked vegetables were analyzed via one-way analysis of variance (ANOVA), followed by Tukey post hoc multiple comparison test. 

Response surface methodology (RSM) was performed using Design-Expert version 13 (Stat-Ease, Inc., Minneapolis, MN, USA). The RSM model was analyzed using the central composite design (CCD). Our study used RSM with CCD to study the effects of three independent variables (cooking time, cooking temperature, and cooking methods) on the dependent variables (the yield of goitrin, BITC, and SFN). The program could help to create the predicted model of relationship among variables and the three-dimension graphs of RSM were generated. Optimization for the best cooking conditions was performed by weighting to minimize goitrin (+++++) as the priority for both cabbage and Chinese kale. For the second priority, we set different weights of BITC and SFN for the two kinds of vegetables. For cabbage, we assigned a higher weight to maximize SFN (++++) than that of BITC (+) because of a slightly higher amount of SFN than BITC in raw cabbage. For Chinese kale, more weight was given to maximize BITC (++++) than SFN (+) due to a much higher amount of BITC than SFN in raw Chinese kale.

## 3. Results and Discussion

### 3.1. Chromatograms and Mass Spectra of Goitrin (GN), Benzyl Isothiocyanate (BITC), and Sulforaphane (SFN)

**Figure 2:**
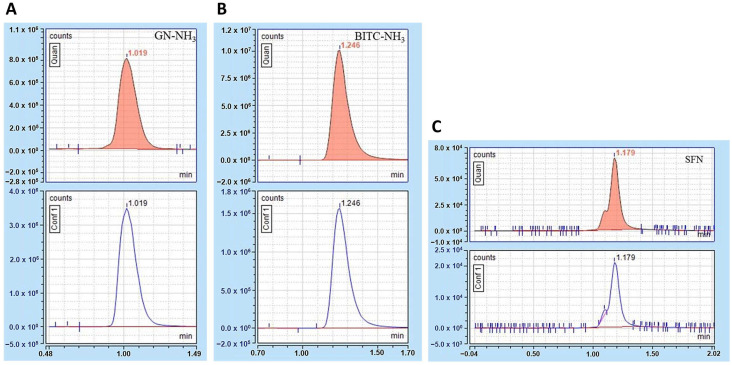
Reference compounds analyzed via LC-MS/MS. Chromatogram of GN-NH_3_ (**A**), BITC-NH_3_ (**B**), and SFN (**C**), for quantified (upper) and confirmation product masses (lower).

The mass-to-charge ratio (*m*/*z*) of the GN-NH_3_ precursor and quantified product mass were 146.85 and 126.49, respectively. A collision energy of 5.25 V was used for the transition. The confirmation product mass for GN-NH_3_ was *m*/*z* 105.97, with a collision energy of 5.34 V for the transition. The retention time of GN-NH_3_ was 1.019 min. The Chromatogram and mass spectra of GN-NH_3_ are shown in Figure 2A.

The mass-to-charge ratio (*m*/*z*) of the BITC-NH_3_ precursor and quantified product mass were 167 and 91.125, respectively. A collision energy of 17.43 V was used for the transition. The confirmation product mass for BITC-NH_3_ was *m*/*z* 65.042 with a collision energy of 39.5 V for the transition. The retention time of BITC was 1.246 min. The Chromatogram and mass spectra of BITC-NH_3_ are shown in Figure 2B.

The mass-to-charge ratio (*m*/*z*) of the SFN precursor and quantified product mass were 178 and 114.07, respectively. A collision energy of 10.48 V was used for the transition. The confirmation product mass for SFN was *m*/*z* 72.04 with a collision energy of 26.57 V for the transition. The retention time of SFN was 1.179 min. The Chromatogram and mass spectra of SFN are shown in Figure 2C. The shouldered peak was found in both standards and samples. Sulforaphane has two isomers: (R)- and (S)-. (R)-Sulforaphane is the major isomer with R configuration at the sulfinyl group. The S-form is an enantiomer of (R)- form with (S)-configuration. The main peak is likely the (R)-form, while the shoulder is likely the S-form. For quantitation, we used the whole-shouldered peak.

Calibration curves of the three compounds showed good linearity with an average R^2^ of 0.9997 for GN-NH_3_, 0.9993 for BITC-NH_3_, and 0.9998 for SFN (Figure S1). Inter-day precision as %RSD for the analyses of GN-NH_3_, BITC-NH_3_, and SFN was 2.28–9.56%, 1.00–2.72%, and 2.44–2.48%, respectively. The mean recovery for the analyses of GN-NH_3_, BITC-NH_3_, and SFN was 85–88%, 82–93%, and 90–94%, respectively. Satisfactory linearity, precision, and recovery were noted when compared to guidelines for standard method performance requirements of AOAC (2016) and ORA Laboratory Manual Volume II of the U.S. FDA [29,30]. The validation data are shown in Table 2.

For measurement of goitrin and BITC, ammonia derivatization was performed with the hexane extract (not with the vegetable samples) before LC/MS-MS analysis. Therefore, the chance of the occurrence of decomposition of glucosinolates by a strong base in this step is low. Or even if it happens, it would happen with all samples in a similar fashion since all samples were treated with ammonia in exactly similar conditions. Therefore, ammonia derivatization should not interfere with the result. We performed a pretest to measure goitrin, BITC, and SFN with or without ammonia derivatization. The result of the pretest showed that goitrin and BITC also can be measured without ammonia derivatization. However, without ammonia derivatization, their ionization patterns in mass spectrometric analysis are not as stable as the ones with ammonia derivatization. This is the reason why we used ammonia derivatization for goitrin and BITC. Unlike BITC and goitrin, the non-derivatized method provided better linearity for SFN than the derivatized method.

### 3.2. Effect of Different Cooking Methods on the Content of Goitrin, BITC, and SFN in Cabbage and Chinese Kale

The yield of goitrin, BITC, and SFN in cabbage after being cooked in different conditions are shown in Table 3 and Figure S2. Uncooked cabbage contained a total of 251.50 ± 59.57 ng/mL of goitrin. A significant decrease in goitrin (*p* < 0.0001) was observed after certain conditions of cooking, i.e., a decrease of 61–81% after blanching at 80 °C for 4–6 min and 100 °C for 2–6 min, a significant decrease of 57–87% after steaming under all tested conditions, and a significant decrease of 58–84% after stir-frying under all test conditions (Table 5). Moreover, the effects of blanching, steaming, and stir-frying in goitrin reduction are not significantly different (*p* > 0.05; Table 3). As shown in Table 5, the best cooking condition for reducing goitrin in cabbage was steaming at the temperature of 80 °C for 4 min, decreasing the percentage change by 87%. Uncooked cabbage contains 108.70 ± 1.08 ng/mL of BITC (Table 3). Blanching, steaming, and stir-frying significantly decreased (*p* < 0.01) BITC content by 6–46%, 12–41%, and 20–38%, respectively when compared with raw samples (Table 5). Based on Table 5, the best cooking condition for preserving BITC in cabbage was blanching at the temperature of 60 °C for 2 min, with the lowest percent loss of 6%. Uncooked cabbage contained 202.08 ± 60.09 ng/mL of SFN. In cabbage, almost all cooking conditions increased SFN by 1.2–16.9-fold (Table 5). Based on Table 5, the best cooking condition for preserving SFN in cabbage was blanching at 60 °C for 2 min, with a 16.9-fold increase (*p* < 0.0001).

The yield of goitrin, BITC, and SFN in Chinese kale are shown in Table 4 and Figure S3. Uncooked Chinese kale had lower levels of goitrin than that of cabbage, with a total amount of goitrin at 82.27 ± 25.23 ng/mL. Blanching of Chinese kale at 60–80 °C for 2–6 min was found to increase goitrin. The result may be explained by an increase in myrosinase activity when heated at 60–80 °C, as reported in a previous study [20]. In contrast, blanching Chinese kale at 100 °C for 6 min significantly decreased goitrin by 73% (*p* < 0.05) when compared with the raw samples (Table 6). However, blanching at 100 °C 4 min, steaming, and stir-frying Chinese kale decreased goitrin by 30%, 1–67%, and 3–66%, respectively, but no significant decrease (*p* > 0.05) was observed when compared with the raw samples (Table 6). Based on Table 6, the best cooking condition for reducing goitrin in Chinese kale was blanching at the temperature of 100 °C for 6 min, with a 73% decrease. Uncooked Chinese kale contains 107.20 ± 2.11 ng/mL of BITC (Table 4). Blanching, steaming, and stir-frying in some conditions did not significantly decrease BITC content (*p* > 0.05) when compared with the raw samples. Instead, some conditions such as stir-frying at 80 °C for 2 min and stir-frying at 100 °C for 2 min significantly increased BITC amount by 36% (*p* < 0.0001) and 19% (*p* < 0.01), respectively (Table 6). The best cooking condition for preserving BITC in Chinese kale was stir-frying at the temperature of 80 °C for 2 min with a 36% increase. Chinese kale contains 9.74 ± 3.86 ng/mL of SFN. While certain conditions increased SFN by 1.1–15.2–fold, some conditions decreased SFN by around 70–80%. The best cooking condition for preserving SFN in Chinese kale was steaming at 60 °C for 6 min, with a 15.2-fold increase (Table 6).

Previously published works only reported changes in progoitrin, the precursor of goitrin, after cooking. Thus, our current study is the first to report the changes in goitrin after cooking. Volden et al. found that the progoitrin in red cabbage was significantly reduced after blanching (78% decrease), boiling (65% decrease), and steaming (54% decrease) [22]. Compared to the previous study, our current work revealed a higher percent decrease of goitrin in cabbage after cooking (81–87%). This distinction may be due to different cooking conditions, analytical methods, and types of cabbage. Wennberg et al. also showed varied percentages of progoitrin reduction after blanching in different cabbage types (47% and 87% decrease in Hekla and Predikant cabbage, respectively [31]. 

For isothiocyanate (ITCs), our results are consistent with a previous study by Wang et al. that showed an increase in ITCs after steaming and stir-frying. Nevertheless, in our current study, the magnitude of SFN increase in cabbage after steaming (15 folds) is higher than those reported in the previous study (1.7 fold) [21]. This may be due to different cooking conditions, analytical methods, and types of vegetables. Consistently, two studies demonstrated that high cooking temperatures can enhance the antioxidant effect of SFN [32,33]. Therefore, heat-induced cooking can also be beneficial for some bioactive compounds.

In our study, the loss of goitrin, BITC, and SFN during different cooking conditions could be explained by various mechanisms: (i) the high-temperature cooking could denature the myrosinase enzyme in *Brassica* vegetables, resulting in the slower conversion of glucosinolates to hydrolysis products (goitrin, BITC, and SFN) during cooking and after mastication [34]; (ii) the loss of glucosinolates at high temperature cooking or leaching into the cooking water would reduce the amount of goitrin and isothiocyanates found in cooked vegetables [21]; (iii) the longer the cooking time, the greater the breakdown of glucosinolates [35]. Moreover, we can indicate that other factors have affected the yield of goitrin and isothiocyanates in our study such as cooking water, sampling samples, food matrix of vegetables, plant species and genotype, plant organ, pre-and -post-harvest factors [36]. These variables may have an impact on the broad SD value in sample vegetables. 

**Table 4:** The yield of goitrin, BITC, and SFN in Chinese kale under different cooking conditions.

Cooking Condition			Goitrin (ng/mL)	BITC (ng/mL)	SFN (ng/mL)
Cooking Methods	Cooking Time (min)	Cooking Temp. (°C)			
Uncooked	−	−	82.3 ± 25.2 ^abc^	107.2 ± 2.1 ^cde^	9.7 ± 3.9 ^g^
Blanching	2	60	84.0 ± 1.0 ^abcd^	108.4 ± 0.4 ^bcde^	55.1 ± 8.7 ^cde^
	4	60	109.5 ± 2.8 ^a^	102.5 ± 0.2 ^cdefg^	19.6 ± 1.6 ^fg^
	6	60	112.4 ± 3.7 ^a^	94.5 ± 0.4 ^defghi^	10.7 ± 0.6 ^g^
Blanching	2	80	104.5 ± 34.6 ^a^	94.7 ± 0.0 ^defghi^	61.5 ± 5.1 ^cd^
	4	80	96.6 ± 1.7 ^ab^	87.7 ± 0.4 ^fghi^	2.7 ± 0.2 ^g^
	6	80	86.3 ± 1.9 ^abcd^	82.1 ± 0.4 ^hi^	2.4 ± 0.1 ^g^
Blanching	2	100	90.4 ± 1.5 ^abc^	93.7 ± 0.2 ^defghi^	26.0 ± 2.9 ^efg^
	4	100	57.4 ± 12.1 ^abcd^	90.0 ± 0.4 ^efghi^	3.6 ± 0.2 ^g^
	6	100	22.4 ± 4.3 ^d^	85.3 ± 0.4 ^ghi^	2.8 ± 0.2 ^g^
Steaming	2	60	33.3 ± 1.1 ^bcd^	118.0 ± 33.3 ^bc^	4.9 ± 0.2 ^g^
	4	60	85.3 ± 1.5 ^abcd^	92.2 ± 0.0 ^efghi^	26.8 ± 0.7 ^efg^
	6	60	58.9 ± 26.3 ^abcd^	81.6 ± 0.2 ^i^	148.4 ± 6.5 ^a^
Steaming	2	80	93.2 ± 19.0 ^ab^	106.1 ± 0.2 ^cdef^	5.4 ± 0.7 ^g^
	4	80	81.8 ± 38.0 ^abcd^	106.3 ± 4.9 ^cdef^	104.6 ± 7.2 ^b^
	6	80	113.2 ± 2.2 ^a^	95.8 ± 0.0 ^defghi^	3.5 ± 0.5 ^g^
Steaming	2	100	56.1 ± 17.3 ^abcd^	95.9 ± 0.2 ^defghi^	23.3 ± 30.6 ^fg^
	4	100	62.2 ± 26.5 ^abcd^	85.4 ± 0.2 ^ghi^	4.4 ± 0.2 ^g^
	6	100	27.4 ± 2.9 ^cd^	82.7 ± 0.2 ^ghi^	3.5 ± 0.1 ^g^
Stir-frying	2	60	27.6 ± 3.5 ^cd^	102.4 ± 0.4 ^cdefg^	31.4 ± 11.0 ^defg^
	4	60	60.8 ± 54.9 ^abcd^	113.1 ± 0.4 ^bcd^	14.9 ± 1.9 ^fg^
	6	60	91.8 ± 4.6 ^abc^	109.1 ± 0.0 ^bcde^	16.8 ± 9.5 ^fg^
Stir-frying	2	80	33.1 ± 4.8 ^bcd^	145.6 ± 0.4 ^a^	43.7 ± 2.2 ^cdef^
	4	80	39.9 ± 38.0 ^bcd^	81.1 ± 0.2 ^i^	3.4 ± 0.3 ^g^
	6	80	55.3 ± 9.1 ^abcd^	81.6 ± 0.4 ^i^	1.9 ± 0.1 ^g^
Stir-frying	2	100	66.1 ± 31.4 ^abcd^	127.3 ± 0.2 ^ab^	63.7 ± 39.1 ^c^
	4	100	79.7 ± 3.0 ^abcd^	101.7 ± 0.2 ^cdefgh^	6.0 ± 0.1 ^g^
	6	100	66.3 ± 3.2 ^abcd^	87.0 ± 0.2 ^fghi^	3.0 ± 0.2 ^g^

The table shows mean ± SD (n = 3 for each cooking condition, n = 6 for uncooked). The superscript letter ^a–i^ indicates the results of statistical analyses using One-way ANOVA with Tukey’s multiple comparison test. The same letter represents no significant difference for pair-wise comparison between conditions (*p* ≥ 0.05). The different letter represents a significant difference for pair-wise comparison between conditions (*p* < 0.05).

**Table 5:** The mean fold changes and percentage change (%) of goitrin, BITC, and SFN in cabbage under different cooking conditions compared with uncooked vegetables.

Cooking Condition			Mean of Fold Change/Percent Change (%), Compared to Uncooked Vegetables		
Cooking Methods	Cooking Time (min)	Cooking Temp. (°C)	Goitrin	BITC	SFN
Uncooked	−	−	100%	100%	100%
Blanching	2	60	1.2/+18%	0.9/−6%	16.9/+1588%
	4	60	0.8/−17%	0.8/−18%	3.4/+245%
	6	60	0.6/−40%	0.8/−21%	1.7/+73%
Blanching	2	80	0.8/−21%	0.9/−13%	4.2/+321%
	4	80	0.3/−71%	0.9/−10%	2.0/+95%
	6	80	0.4/−61%	0.5/−46%	2.5/+152%
Blanching	2	100	0.2/−79%	0.8/−22%	2.5/+151%
	4	100	0.2/−81%	0.6/−44%	0.3/−66%
	6	100	0.3/−70%	0.7/−34%	0.5/−48%
Steaming	2	60	0.4/−57%	0.7/−32%	1.2/+20%
	4	60	0.3/−68%	0.8/−17%	1.4/+36%
	6	60	0.2/−79%	0.8/−21%	5.1/+409%
Steaming	2	80	0.2/−75%	0.8/−16%	0.7/−26%
	4	80	0.1/−87%	0.7/−34%	4.0/+296%
	6	80	0.2/−82%	0.6/−41%	2.2/+117%
Steaming	2	100	0.2/−82%	0.7/−28%	0.6/−36%
	4	100	0.2/−82%	0.9/−12%	3.3/+235%
	6	100	0.2/−78%	0.7/−29%	1.6/+55%
Stir-frying	2	60	0.3/−73%	0.8/−20%	5.7/+474%
	4	60	0.2/−84%	0.7/−30%	2.5/+151%
	6	60	0.2/−84%	0.7/−31%	2.3/+128%
Stir-frying	2	80	0.2/−77%	0.7/−28%	1.2/+25%
	4	80	0.2/−79%	0.6/−38%	2.6/+156%
	6	80	0.2/−81%	0.7/−27%	2.8/+177%
Stir-frying	2	100	0.4/−59%	0.7/−28%	1.6/+58%
	4	100	0.3/−66%	0.7/−32%	2.4/+136%
	6	100	0.4/−58%	0.6/−36%	2.3/+126%

The table shows mean fold changes and percentage change (%), which have the minus sign (−) and the plus sign (+) denoting decreasing and increasing levels, respectively, in each substance under different cooking conditions compared with uncooked. Minus sign (−) under cooking time and temperature represents no cooking.

**Table 6:** The mean fold changes and percentage change (%) of goitrin, BITC, and SFN in Chinese kale under different cooking conditions compared with uncooked condition.

Cooking Condition			Mean of Fold Change/Percent Change (%), Compared to Uncooked Vegetables		
Cooking Methods	Cooking Time (min)	Cooking Temp. (°C)	Goitrin	BITC	SFN
Uncooked	−	−	−	−	−
Blanching	2	60	1.0/+2%	1.0/+1%	5.7/+466%
	4	60	1.3/+33%	1.0/−4%	2.0/+101%
	6	60	1.4/+37%	0.9/−12%	1.1/+10%
Blanching	2	80	1.3/+27%	0.9/−12%	6.3/+531%
	4	80	1.2/+17%	0.8/−18%	0.3/−72%
	6	80	1.0/+5%	0.8/−23%	0.2/−75%
Blanching	2	100	1.1/+10%	0.9/−13%	2.7/+167%
	4	100	0.7/−30%	0.8/−16%	0.4/−63%
	6	100	0.3/−73%	0.8/−20%	0.3/−72%
Steaming	2	60	0.4/−60%	1.1/+10%	0.5/−50%
	4	60	1.0/+4%	0.9/−14%	2.8/+176%
	6	60	0.7/−28%	0.8/−24%	15.2/+1424%
Steaming	2	80	1.1/+13%	1.0/−1%	0.6/−45%
	4	80	1.0/−1%	1.0/−1%	10.7/+974%
	6	80	1.4/+38%	0.9/−11%	0.4/−64%
Steaming	2	100	0.7/−32%	0.9/−11%	2.4/+139%
	4	100	0.8/−24%	0.8/−20%	0.5/−55%
	6	100	0.3/−67%	0.8/−23%	0.4/−64%
Stir-frying	2	60	0.3/−66%	1.0/−4%	3.2/+222%
	4	60	0.7/−26%	1.1/+6%	1.5/+53%
	6	60	1.1/+12%	1.0/+2%	1.7/+73%
Stir-frying	2	80	0.4/−60%	1.4/+36%	4.5/+349%
	4	80	0.5/−52%	0.8/−24%	0.4/−65%
	6	80	0.7/−33%	0.8/−24%	0.2/−80%
Stir-frying	2	100	0.8/−20%	1.2/+19%	6.5/+554%
	4	100	1.0/−3%	0.9/−5%	0.6/−39%
	6	100	0.8/−19%	0.8/−19%	0.3/−69%

The table shows mean fold changes and percentage change (%), which have the minus sign (−) and the plus sign (+) denoting decreasing and increasing in each substance under different cooking conditions compared with uncooked. Minus sign (−) under cooking time and temperature represents no cooking.

### 3.3. Optimization of Cooking Methods for Cabbage and Chinese Kale

**Figure 3:**
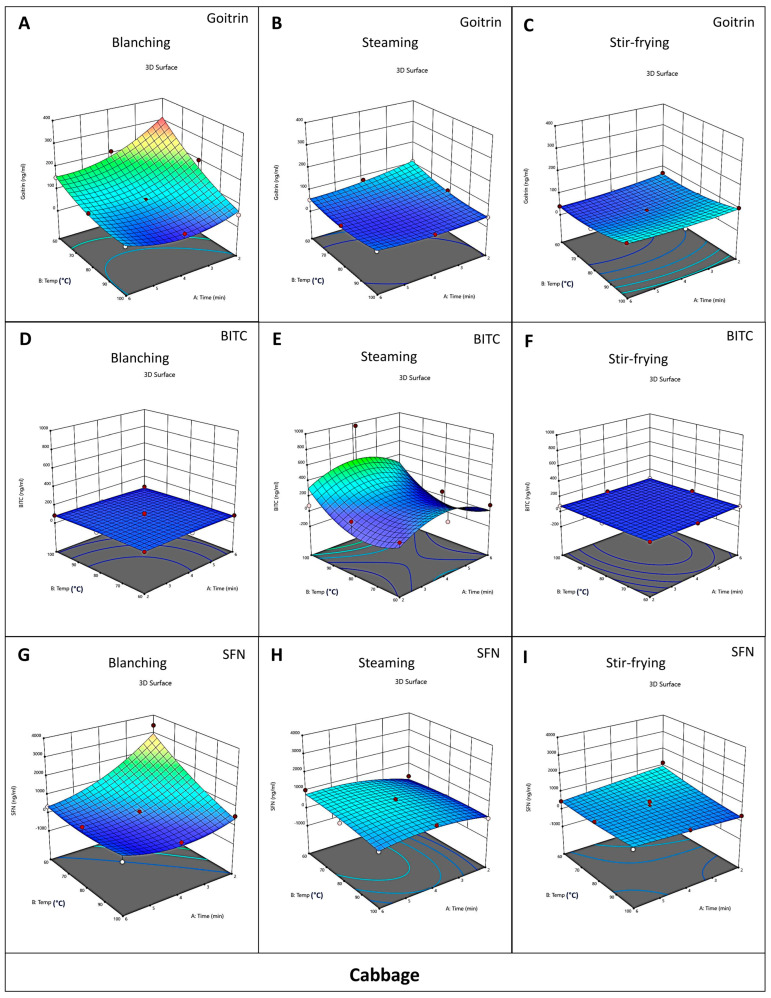
Relationship between cooking factors in cabbage. Three-dimensional (3D) response surface methodology (RSM) diagrams show concentrations of goitrin (**A**–**C**), BITC (**D**–**F**), and SFN (**G**–**I**) in different cooking conditions of cabbage as the function of cooking time, cooking temperature, and cooking methods. Red, yellow, green, and blue areas depict the highest to lowest goitrin, BITC, and SFN concentrations.

**Figure 4:**
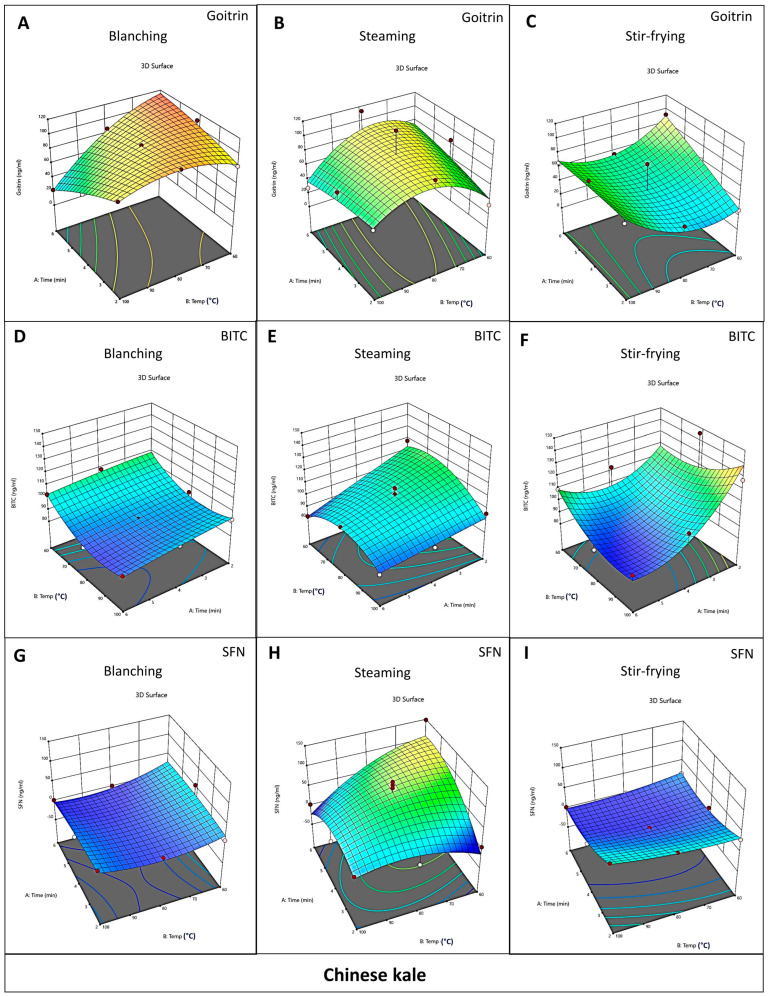
Relationship between cooking factors in Chinese kale. Three-dimensional (3D) response surface methodology (RSM) diagrams show concentrations of goitrin (**A**–**C**), BITC (**D**–**F**), and SFN (**G**–**I**) in different cooking conditions of Chinese kale as the function of cooking time, cooking temperature, and cooking methods. Red, yellow, green, and blue areas depict the highest to lowest goitrin, BITC, and SFN concentrations.

The results of this research demonstrated that the concentrations of goitrin, BITC, and SFN were influenced by cooking temperature, cooking time, and cooking methods. The interactions between these factors were found via ANOVA analysis. To determine which cooking method is the best for reducing goitrin while preserving isothiocyanates in *Brassica* vegetables, response surface methodology (RSM) models were used. The RSM graphs of goitrin (Figure 3A–C and Figure 4A–C), BITC (Figure 3D–F and Figure 4D–F), and SFN (Figure 3G–I and Figure 4G–I) are shown. The warm tone color zones (lowest in blue and maximum in red) represent the components’ optimal values.

In this study, the weights of BITC and SFN in the analyses for cabbage and Chinese kale were set differently. Uncooked cabbage contains a higher amount of SFN than BITC. In contrast, raw Chinese kale has a higher amount of BITC than SFN. Therefore, we provided a higher weight in SFN for cabbage and a higher weight in BITC for Chinese kale.

In cabbage, the response surface analysis (RSA) using ANOVA showed that the concentration of goitrin depended on the cooking temperature, cooking time, and cooking methods, but the concentration of BITC did not significantly depend on those factors. Furthermore, the concentration of SFN in cabbage depended on the cooking temperature and cooking time but not on cooking methods. When the concentrations were weighed after minimizing goitrin (+++++), maximizing BITC (+), and maximizing SFN (++++), the data showed that the best cooking method to reduce goitrin and preserve ITCs in cabbage was steaming at a temperature of 90.92 °C for 4.26 min. (desirability 0.38) (Table 7 and Figure S4).

In Chinese kale, the response surface analysis (RSA) using ANOVA showed that the concentrations of goitrin and SFN depended on cooking methods but not cooking temperature and time. In contrast, the concentration of BITC depended on the cooking temperature and cooking time but not on cooking methods. When the concentrations were weighed after by minimizing goitrin (+++++), maximizing BITC (++++), and maximizing SFN (+), the data showed that the best cooking method to reduce goitrin and preserve ITCs in Chinese kale was stir-frying at a temperature of 84.56 °C for 2 min. (desirability 0.63) (Table 8 and Figure S4). It is worth noting that although blanching Chinese kale at 100 °C for 6 min was the best method to significantly decrease goitrin, such a method also caused a significant loss of BITC and SFN. When RSM analysis was performed by weighing both the reduction in goitrin and preservation of BITC and SFN in Chinese kale, the results suggest that stir-frying was a better method with higher desirability than that of blanching (Table 7 and Table 8).

The optimized conditions for reducing goitrin and preserving isothiocyanates are in decimal digits, and it might be difficult to achieve such values in household cooking. Moreover, due to the limitation of the central composite design in this research, the exact optimized condition obtained from the analysis may not be suitable for recommendation. Thus, a range of values was further identified. Table 7 and Table 8 illustrates the top-ranked cooking conditions with the best desirability values for reducing goitrin and preserving isothiocyanates in Chinese kale and cabbage. For Chinese kale, the best range of optimized cooking conditions with the best desirability values (0.59–0.63) is stir-frying at 60–100 °C for 2 min (Table 8). For cabbage, since the best desirability value is still quite low (0.38), we considered the data analyzed via RSM (Table 7) together with the data analyzed with ANOVA (Table 5). The results showed that steaming cabbage at 80 °C and 100 °C for 4 min still could reduce goitrin and also preserve BITC and SFN. The best cooking of cabbage obtained from RSM was steaming at 90.92 °C for 4.26 min.

Based on these findings, we suggest that the optimized household cooking methods for preserving isothiocyanates and reducing goitrin are steaming cabbage at 80–100 °C for 4 min, and stir-frying Chinese kale at 60–100 °C for 2 min.

### 3.4. Strengths and Limitations of This Research

This study has several strengths. First, several affecting factors have been controlled. Cooking was performed in a home kitchen using the very same induction cooker for all cooking methods (blanching, steaming, and stir-frying), ensuring real household cooking conditions and fair control of the cooking apparatus. The temperature of the cooker is also controlled with a digital system, allowing the user to adjust the temperature precisely. The water used in all conditions was from the same source. In addition, the same bulb of vegetables has been divided evenly into four parts for all cooking conditions and raw samples. Since our sample contains a mixture of leaf and stem, we ground the cooked or uncooked samples before collection for analysis. This is to ensure the homogeneity of collected samples. Second, this is the first study to report the changes in goitrin after cooking, while previous studies only reported changes in progoitrin, the precursor of goitrin, after cooking. Thus, the findings of this study can guide which cooking conditions may help to reduce goitrin. Third, this work studied two *Brassica* vegetables (cabbage and Chinese kale), which are commonly consumed in many countries around the world. Therefore, the insight from this work may have broad application.

There were some limitations of this study. First, the yield of goitrin, BITC, and SFN in vegetable samples had wide standard deviation (SD) values. Though we tried our best to control the possible confounding factors, there are still some variables we cannot control. Since we used manual household cooking in the open air, the whole pieces of vegetables may not be evenly cooked (unlike the factory cooking in the closed system). The second limitation is that we set the temperature of cooking from an induction cooker and did not directly measure the temperature on the vegetables during cooking. Therefore, the insight from this work is only applicable for household cooking, and may not be practical for industrial use. Future studies for closed-system cooking are warranted for industrial purposes. The third limitation is the design of RSM analysis. Since the cooking method is not in numerical or ordinal scales and cannot make three levels of factorial experiments, the Box–Behnken design (BBD) cannot be used in this study. A central composite design was used instead. For the last limitation of this study, we used cooking water for the stir-frying method in our study because other cooking methods (blanching and steaming) also used water. Nevertheless, water stir-frying is not as commonly used as oil stir-frying. Future works are warranted to study the best condition of oil-based methods, such as stir-frying, deep frying, and grilling with oil, to reduce goitrin while preserving ITCs. In addition, cooking methods to reduce goitrin while preserving other ITCs with chemo-preventive effects such as allyl isothiocyanate (AITC), phenethyl isothiocyanate (PEITC), 3-Butenyl Isothiocyanate (MTBITC), warrants further studies. 

## 4. Conclusions

Using LC-MS/MS for the determination of goitrin, BITC, and SFN together with the response surface methodology, we found that cooking temperature, cooking time, and cooking methods were the factors that influenced the levels of goitrin and isothiocyanates (BITC and SFN) in cabbage and Chinese kale. The optimum cooking method for preserving isothiocyanates and reducing goitrin is steaming cabbage at 80–100 °C for 4 min and stir-frying Chinese kale at 60–100 °C for 2 min. Such cooking methods may help to maintain cancer chemopreventive compounds (ITCs) and remove the anti-thyroid compound (goitrin). Eventually, it may help to reduce the risk of cancer in the long term.

## Figures and Tables

**Table 1:** Cooking water, cooking temperature, and cooking time under different cooking methods for cabbage and Chinese kale.

	Cooking Methods	Weight (g)	Water Volume (mL)	Cooking Time (min)			Cooking Temp. (°C)		
Cabbage	Blanching	150	400	2	4	6	60	80	100
	Steaming	150	400	2	4	6	60	80	100
	Stir-frying	150	400	2	4	6	60	80	100
Chinese kale	Blanching	150	400	2	4	6	60	80	100
	Steaming	150	400	2	4	6	60	80	100
	Stir-frying	150	400	2	4	6	60	80	100

**Table 2:** Validation data for LC-MS/MS analysis of goitrin, BITC, and SFN in cabbage and Chinese kale.

Analyte	R^2^	Mean Recovery (%)			Inter-Day Precision (%RSD)			
		Cabbage	Chinese Kale	Expected Criteria ^a^	Vegetable Samples	Expected Criteria ^b^	Standard 1 ng/mL	Expected Criteria ^c^
Goitrin	0.9997	88	85	60–115	2.28	21	9.56	30
BITC	0.9993	93	82	60–115	1.00	21	2.72	30
SFN	0.9998	94	90	60–115	2.44	21	2.48	30

^a^ Expected recovery at 10 ng/mL from AOAC Peer-Verified Methods Program (2016). ^b^ Expected precision (repeatability) at 10 ng/mL from AOAC Peer-Verified Methods Program (2016). ^c^ Expected precision (repeatability) at 1 ng/mL from AOAC Peer-Verified Methods Program (2016).

**Table 3:** The yield of goitrin, BITC, and SFN in cabbage under different cooking conditions.

Cooking Condition			Goitrin (ng/mL)	BITC (ng/mL)	SFN (ng/mL)
Cooking Methods	Cooking Time (min)	Cooking Temp. (°C)			
Uncooked	−	−	251.5 ± 59.6 ^a^	108.7 ± 1.1 ^a^	202.8 ± 60.1 ^f^
Blanching	2	60	296.5 ± 128.4 ^a^	102.6 ± 1.1 ^ab^	3423.0 ± 1014.0 ^a^
	4	60	209.8 ± 7.6 ^ab^	88.8 ± 0.8 ^bcdefgh^	698.9 ± 162.6 ^bcdef^
	6	60	152.1 ± 85.1 ^bcd^	86.0 ± 1.4 ^bcdefghi^	350.5 ± 101.0 ^def^
Blanching	2	80	199.4 ± 40.7 ^abc^	94.8 ± 9.0 ^abcde^	853.0 ± 231.7 ^bcd^
	4	80	72.5 ± 13.4 ^de^	97.8 ± 10.7 ^abc^	395.5 ± 24.5 ^cdef^
	6	80	97.6 ± 27.5 ^bcde^	58.5 ± 0.9 ^m^	510.1 ± 46.7 ^bcdef^
Blanching	2	100	53.2 ± 2.4 ^de^	84.4 ± 0.4 ^cdefghij^	509.6 ± 31.9 ^bcdef^
	4	100	48.2 ± 5.6 ^de^	60.8 ± 20.9 ^lm^	68.0 ± 15.7 ^f^
	6	100	76.3 ± 8.5 ^de^	71.9 ± 0.6 ^hijklm^	104.6 ± 49.5 ^f^
Steaming	2	60	108.5 ± 26.9 ^bcde^	74.3 ± 0.4 ^ghijklm^	242.9 ± 59.5 ^def^
	4	60	80.9 ± 5.8 ^de^	89.9 ± 0.2 ^bcdefg^	276.7 ± 26.8 ^def^
	6	60	53.4 ± 4.5 ^de^	86.0 ± 9.7 ^bcdefghi^	1033.0 ± 208.1 ^bc^
Steaming	2	80	62.8 ± 11.7 ^de^	91.8 ± 6.3 ^bcdef^	150.8 ± 7.3 ^ef^
	4	80	33.7 ± 0.6 ^e^	72.2 ± 0.2 ^hijklm^	802.6 ± 46.4 ^bcde^
	6	80	45.5 ± 8.1 ^de^	64.3 ± 0.2 ^klm^	440.5 ± 87.3 ^cdef^
Steaming	2	100	45.2 ± 15.3 ^de^	77.8 ± 0.0 ^fghijkl^	130.6 ± 16.5 ^ef^
	4	100	45.0 ± 1.7 ^de^	95.5 ± 0.2 ^abcd^	679.3 ± 36.6 ^bcdef^
	6	100	55.3 ± 4.8 ^de^	77.4 ± 0.4 ^fghijkl^	314.6 ± 62.8 ^def^
Stir-frying	2	60	67.9 ± 16.3 ^de^	86.6 ± 0.2 ^bcdefghi^	1164.0 ± 21.5 ^b^
	4	60	40.8 ± 6.2 ^de^	76.6 ± 0.6 ^fghijkl^	508.2 ± 134.1 ^bcdef^
	6	60	39.6 ± 14.0 ^de^	75.4 ± 7.9 ^fghijklm^	462.2 ± 66.3 ^cdef^
Stir-frying	2	80	57.6 ± 0.4 ^de^	78.1 ± 0.0 ^efghijk^	253.0 ± 37.8 ^def^
	4	80	52.9 ± 1.4 ^de^	67.5 ± 0.2 ^jklm^	519.5 ± 247.9 ^bcdef^
	6	80	48.1 ± 14.2 ^de^	79.8 ± 0.2 ^defghijk^	561.5 ± 81.5 ^bcdef^
Stir-frying	2	100	102.4 ± 15.6 ^bcde^	78.4 ± 0.4 ^efghijk^	319.7 ± 26.4 ^def^
	4	100	86.3 ± 2.1 ^cde^	73.4 ± 0.0 ^ghijklm^	478.8 ± 37.4 ^cdef^
	6	100	105.1 ± 2.8 ^bcde^	69.9 ± 0.2 ^ijklm^	458.5 ± 195.6 ^cdef^

The table shows mean ± SD (n = 3 for each cooking condition, n = 6 for uncooked). The superscript letter ^a–m^ indicates the results of statistical analyses using One-way ANOVA with Tukey’s multiple comparison test. The same letter represents no significant difference for pair-wise comparison between conditions (*p* ≥ 0.05). The different letter represents a significant difference for pair-wise comparison between conditions (*p* < 0.05).

**Table 7:** The rank of conditions with the best desirability values of cabbage.

Number	Time (min)	Temp. (°C)	Cooking Method	Goitrin (ng/mL)	BITC (ng/mL)	SFN (ng/mL)	Desirability
1	4.26	90.92	Steaming	33.45	278.72	681.39	0.38
2	2.71	60.00	Stir-frying	56.30	80.10	875.54	0.29
3	2.68	60.00	Stir-frying	56.71	80.39	879.95	0.29
4	2.63	60.00	Stir-frying	57.29	80.67	885.96	0.29
5	2.87	60.00	Stir-frying	54.50	79.11	855.86	0.29
6	2.96	60.00	Stir-frying	53.54	78.60	844.90	0.29
7	4.09	60.00	Stir-frying	43.96	74.60	704.61	0.28
8	4.30	60.00	Stir-frying	42.66	74.35	677.52	0.27
9	4.70	60.00	Stir-frying	40.68	74.32	626.40	0.27
10	2.42	92.81	Blanching	78.21	88.59	472.44	0.18
11	5.89	98.65	Blanching	73.32	63.37	343.13	0.14

The table shows 11 solutions found for 3 combinations of categoric factor levels in cabbage from Design-Expert version 13. Grey shade represents condition with the best desirability value.

**Table 8:** The rank of conditions with the best desirability values of Chinese kale.

Number	Time (min)	Temp. (°C)	Cooking Method	Goitrin (ng/mL)	BITC (ng/mL)	SFN (ng/mL)	Desirability
1	2.00	84.56	Stir-frying	31.90	121.22	45.26	0.63
2	2.00	84.78	Stir-frying	32.26	121.39	45.44	0.63
3	2.00	84.95	Stir-frying	32.54	121.51	45.57	0.63
4	2.00	85.10	Stir-frying	32.80	121.63	45.70	0.63
5	2.00	84.01	Stir-frying	31.02	120.81	44.82	0.63
6	2.00	83.77	Stir-frying	30.65	120.65	44.64	0.63
7	2.00	85.86	Stir-frying	34.12	122.23	46.33	0.63
8	2.00	87.39	Stir-frying	37.01	123.54	47.67	0.63
9	2.00	62.12	Stir-frying	26.49	117.22	35.37	0.61
10	2.00	61.75	Stir-frying	26.92	117.37	35.34	0.61
11	2.00	61.11	Stir-frying	27.70	117.64	35.31	0.61
12	2.00	60.25	Stir-frying	28.84	118.04	35.28	0.61
13	2.00	97.00	Stir-frying	61.74	134.44	57.77	0.59
14	2.72	60.00	Steaming	49.52	107.39	30.35	0.46
15	2.74	60.00	Steaming	49.72	107.24	31.40	0.46
16	2.78	60.00	Steaming	50.13	106.93	33.55	0.46
17	2.66	60.00	Steaming	48.91	107.85	27.12	0.46
18	2.80	60.00	Steaming	50.39	106.73	34.89	0.46
19	2.00	60.00	Blanching	86.26	109.12	21.77	0.31
20	6.00	60.00	Blanching	86.45	109.10	21.90	0.31
21	2.32	60.29	Blanching	86.73	108.72	21.26	0.31
22	6.00	63.22	Stir-frying	80.98	104.01	15.86	0.30
23	2.32	60.00	Blanching	89.59	108.80	24.01	0.30
24	4.11	60.00	Stir-frying	62.49	97.11	8.11	0.29
25	3.80	100.00	Blanching	62.41	90.65	15.34	0.26
26	3.82	100.00	Blanching	62.22	90.62	15.28	0.26
27	3.84	100.00	Blanching	61.81	90.56	15.14	0.26
28	3.91	100.00	Blanching	60.67	90.39	14.76	0.26
29	4.35	100.00	Blanching	53.14	89.23	12.00	0.25
30	4.30	60.00	Blanching	106.45	105.99	32.20	0.20

The table shows 30 solutions found for three combinations of categoric factor levels in Chinese kale from Design-Expert version 13. Grey shade represents condition with the best desirability value.

## Data Availability

Data is contained within the article or Supplementary Material.

## Supplementary Materials

The following supporting information can be downloaded at: https://www.mdpi.com/article/10.3390/foods12193647/s1. Figure S1: Average calibration curves of GN-NH_3_ (A), BITC-NH_3_ (B), and SFN (C). Figure S2. Goitrin and isothiocyanate content in cabbage samples after cooking conditions: (A) goitrin content; (B) benzyl-isothiocyanate (BITC) content; (C) sulforaphane (SFN) content. Figure S3. Goitrin and isothiocynate content in Chinese kale samples after cooking conditions: (A) goitrin content; (B) benzyl-isothiocyanate (BITC) content; (C) sulforaphane (SFN) content. Figure S4. The optimal condition of cabbage (A) and Chinese kale (B) for reducing goitrin while preserving isothiocyanates (BITC and SFN) in *Brassica* vegetables through the response surface methodological (RSM).
