# Supplementary material for: Cooking Methods for Preserving Isothiocyanates and Reducing Goitrin in Brassica Vegetables
Source: Foods. 2023 Oct 2;12(19):3647. doi: 10.3390/foods12193647 (PMC10573036; doi:10.3390/foods12193647)
Supplement: Supplementary file 1 [file foods-12-03647-s001.zip › foods-2582046-supplementary.pdf]

**Supplementary Material**

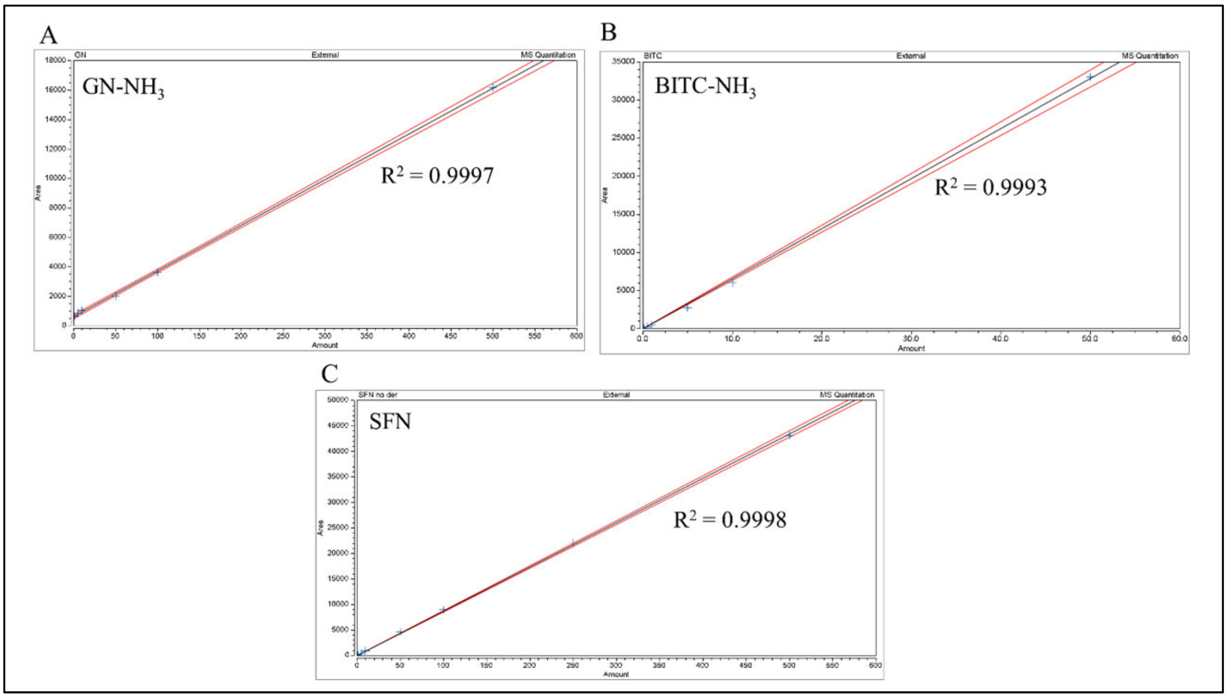


**Figure S1***:* Average calibration curves of GN-$NH_3$ (A), BITC-$NH_3$ (B), and SFN (C) were generated by the linear plots between areas under the curves of the quantitative product ions and the concentrations of standard solutions. $R^2$ values were obtained from linear regression.

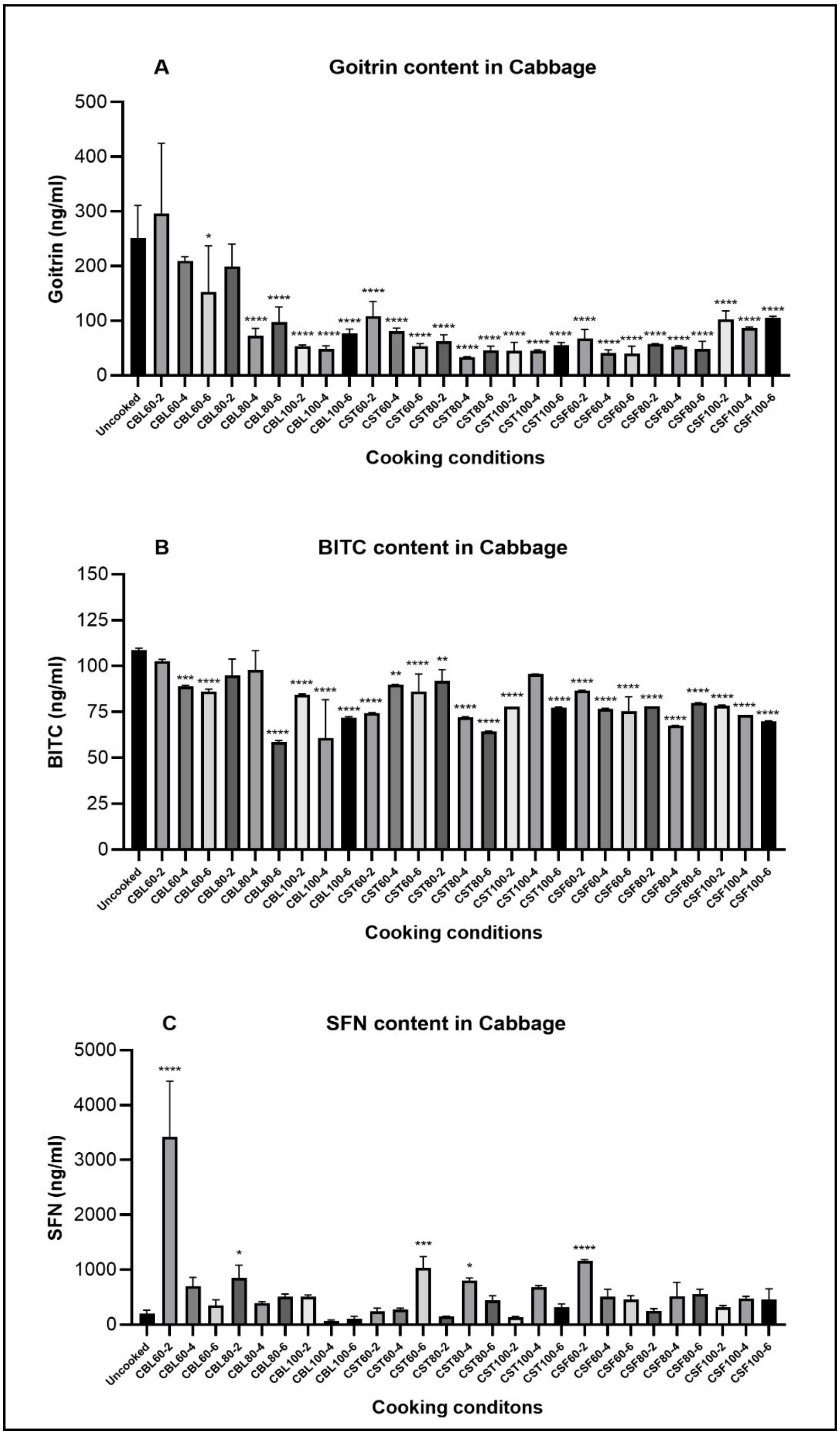


**Figure S2.** Goitrin and isothiocyanate content in cabbage samples after cooking conditions: (A) goitrin content; (B) benzyl-isothiocyanate (BITC) content; (C) sulforaphane (SFN) content. Data are ng/ml (mean ± SD). Significant: *, $p < 0.05$; **, $p < 0.01$; ***, $p < 0.001$; ****, $p < 0.0001$ concerning goitrin or isothiocyanate content of cabbage before cooking. C = cabbage, BL = blanching, ST = steaming, SF = Stir-frying; time = 2, 4, 6 min; temp. = 60, 80, 100 °C.

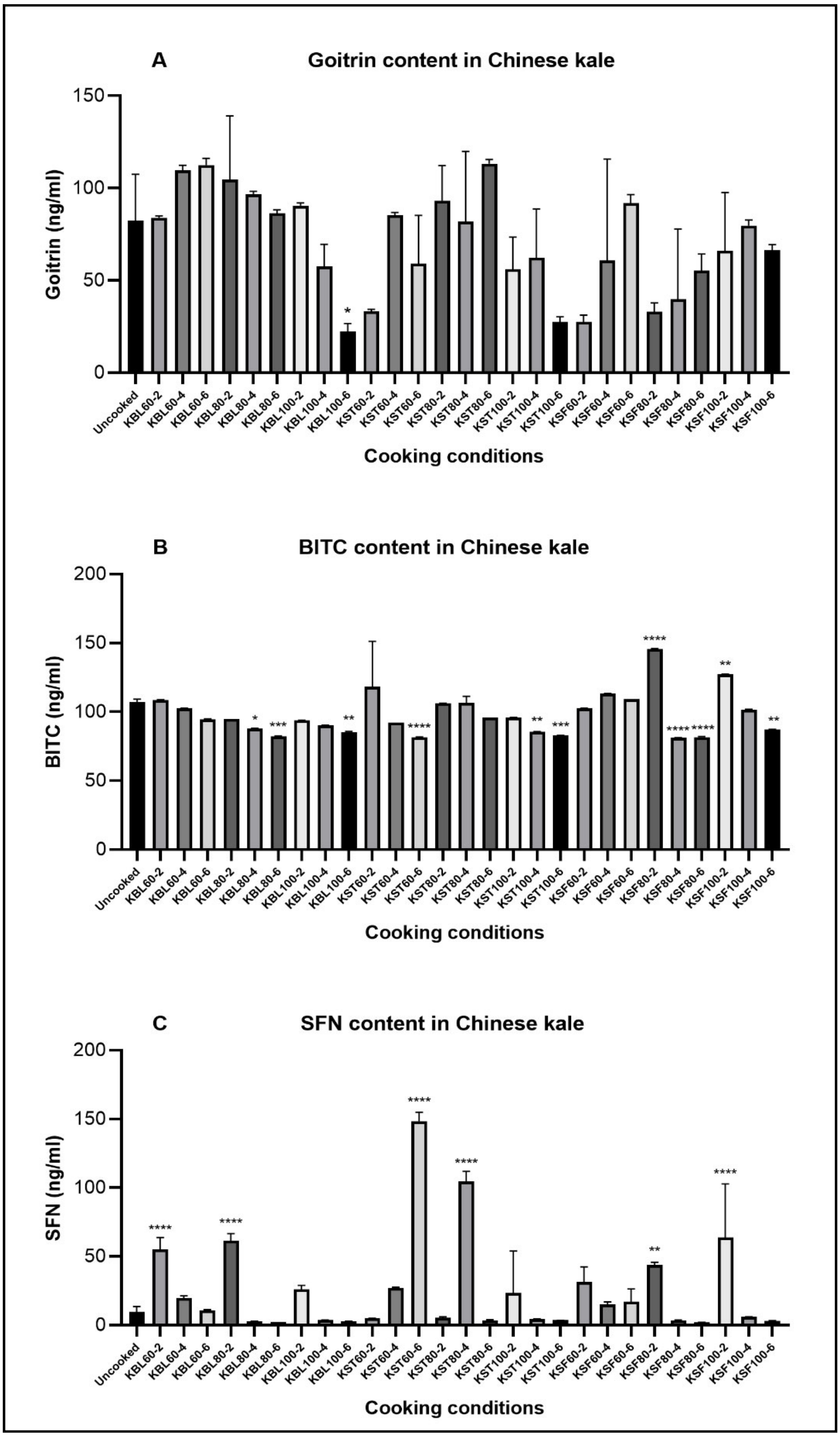


**Figure S3**. Goitrin and isothiocyanates content in Chinese kale samples after cooking conditions: (A) goitrin content; (B) benzyl-isothiocyanate (BITC) content; (C) sulforaphane (SFN) content. Data are ng/ml (mean ± SD). Significant: *, $p < 0.05$; **, $p < 0.01$; ***, $p < 0.001$; ****, $p < 0.0001$ concerning goitrin or isothiocyanate content of Chinese kale before cooking. K = Chinese kale, BL = blanching, ST = steaming, SF = Stir-frying; time = 2, 4, 6 min; temp. = 60, 80, 100 °C.

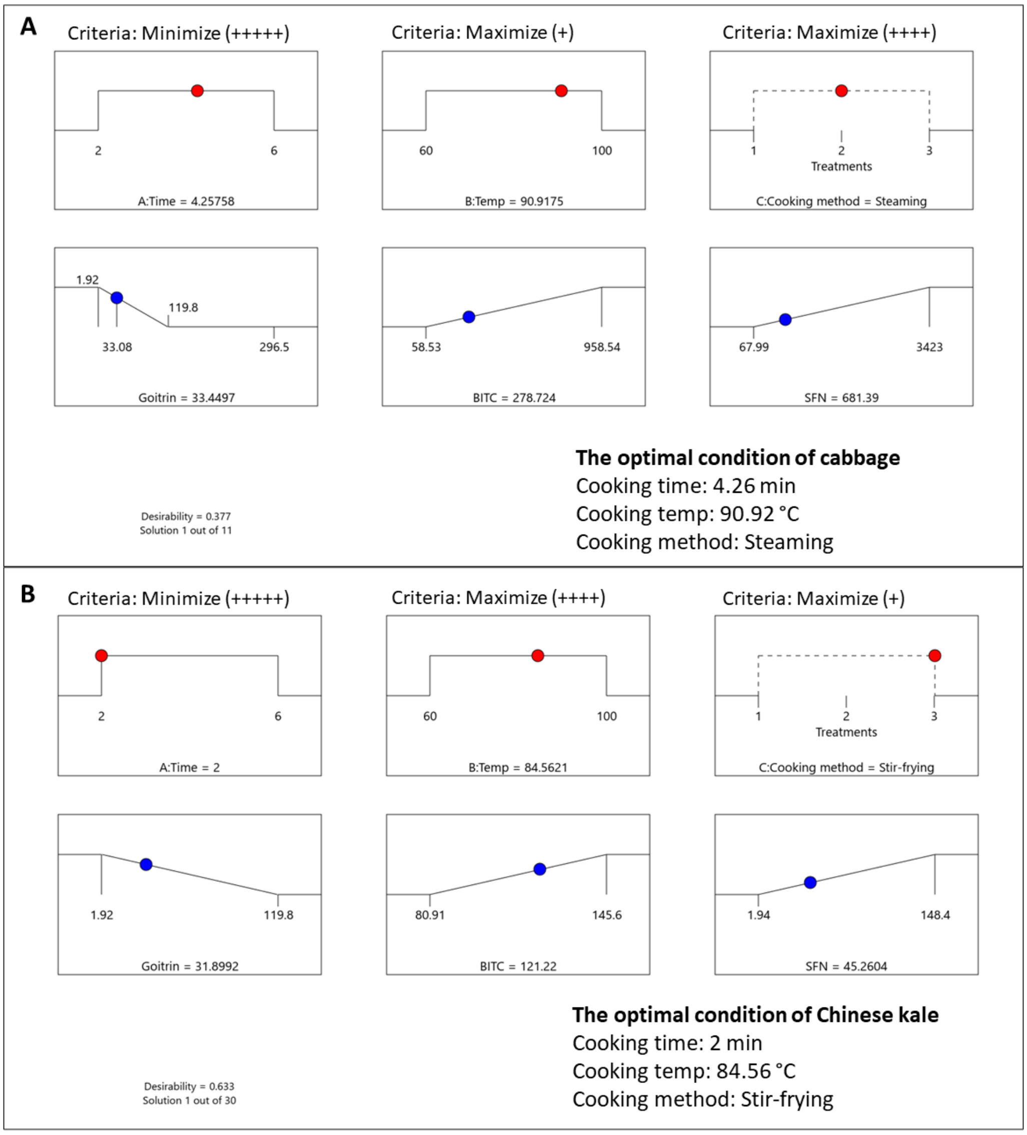


**Figure S4.** The optimal condition of cabbage (A) and Chinese kale (B) for reducing goitrin while preserving isothiocyanates (BITC and SFN) in Brassica vegetables through the response surface methodological (RSM) approach by weighing: minimize of goitrin (+++++), maximize of BITC (+), and maximize of SFN (++++) in cabbage while weighting: minimize of goitrin (+++++), maximize of BITC (++++), and maximize of SFN (+).
